# Variable Endoscopist performance in proximal and distal adenoma detection during colonoscopy: a retrospective cohort study

**DOI:** 10.1186/s12876-018-0800-4

**Published:** 2018-05-30

**Authors:** Paul James, Mehdi Hegagi, Mae Hegagi, Lilia Antonova, Alaa Rostom, Catherine Dube, Sanjay Murthy, Rakesh Goel, Avijit Chatterjee

**Affiliations:** 1 Department of Medicine, The University of Ottawa, Ottawa, Canada; 2 Ottawa Hospital Research Institute, The University of Ottawa, Ottawa, Canada; 3 Department of Medicine, University Health Network, University of Toronto, 200 Elizabeth Street, Room 9N-981, Toronto, ON M5G 2C4 Canada

**Keywords:** Adenoma detection rate, Adenoma, Colonoscopy, Colorectal cancer

## Abstract

**Background:**

Adenoma Detection Rate (ADR) is a validated colonoscopy quality indicator. In addition to overall ADR, Distal and Proximal Adenoma Detection Rates may provide important colonoscopy quality information. The goal of this study is to determine the association between distal and proximal adenoma detection (AD) and to identify factors contributing to overall, distal, and proximal AD.

**Methods:**

This is a retrospective cohort study of patients with a noted family history of CRC or positive fecal occult blood test who underwent a screening colonoscopy at a regional colorectal cancer (CRC) screening center between May 2009 and December 2011. Data regarding patient demographics, procedure details, endoscopist characteristics and polyp histology were captured. The main outcomes measured were overall, distal, and proximal AD.

**Results:**

1907 patients were included. The median age was 60 years and 42% were male. Endoscopist median overall ADR was 25% (30% male, 21% female). Endoscopist distal ADR was only modestly associated with their proximal ADR (Spearman Rank: 0.51 *p* = 0.11). Highest overall ADR (29 to 45%) was found for endoscopists whose distal and proximal ADRs were above the group median. In multivariate analysis, factors associated with overall, distal, and proximal AD included age, sex, and endoscopist practicing experience.

**Conclusion:**

Inclusion of distal and proximal ADRs, in addition to overall ADR, in colonoscopy quality assessment provides the more accurate feedback on endoscopist performance.

## Background

Colonoscopy is considered the most effective method for the identification and removal of adenomatous polyps [1]. However, multiple studies have shown significant polyp and adenoma miss rates for colonoscopies [2], as well as lower than expected reduction in mortality for proximal colorectal cancers [3]. These factors have highlighted the necessity for effective quality indicators to monitor and advance colon cancer screening programs.

The quality indicator currently recommended by the American Society for Gastrointestinal Endoscopy is Adenoma Detection Rate (ADR) [4, 5]. ADR is defined as the proportion of colonoscopies where at least one adenoma is found [6]. An endoscopist’s ADR has been shown to be associated with overall patient risk of interval colorectal cancer (colorectal cancer diagnosed within a few years of colonoscopy) [7, 8], risk of distal interval colorectal cancer [9], and risk of fatal interval colorectal cancer [8].

Little is known about the rate of adenoma detection in specific segments of the colon. Lesions in the distal and proximal colon have been shown to adhere to differing patterns of development. Specifically, sessile serrated adenomas are primarily observed in the proximal colon. These lesions are more difficult to detect and resect and have been proposed to follow a unique serrated pathway that progresses to carcinogenesis more rapidly than the APC-linked pathway of other adenomas [10–12]. Whether this variablity in adenoma presentation in different segments of the colon is reflected in variable rates of adenoma detection between segments, remains to be determined. Recent publications and guidelines have focused on identifying adenomas in the proximal colon. To our knowledge, only two studies to date have examined adenoma detection rates in the distal colon [13, 14]. These have produced inconsistent results. Whereas Boroff and colleagues observed a lower inter-operator (between endoscopists) ADR for the distal colon, as compared to the proximal colon [13], Schramm and colleagues observed higher distal ADR [14]. Intra-operator (for the same endoscopist) differences for distal and proximal detection have not been previously assessed.

Identifying variability in an endoscopist’s detection of distal vs proximal adenomas offers an opportunity to enhance performance assessment and improve colonoscopy quality. The aims of the study presented here are to 1) determine if intra-operator differences exist in distal and proximal ADR and 2) identify factors that predict overall, distal, and proximal adenoma detection.

## Methods

### Ethics approval

This retrospective study protocol was approved by the Ottawa Hospital Research Ethics Board.

### Study protocol

**Fig. 1:**
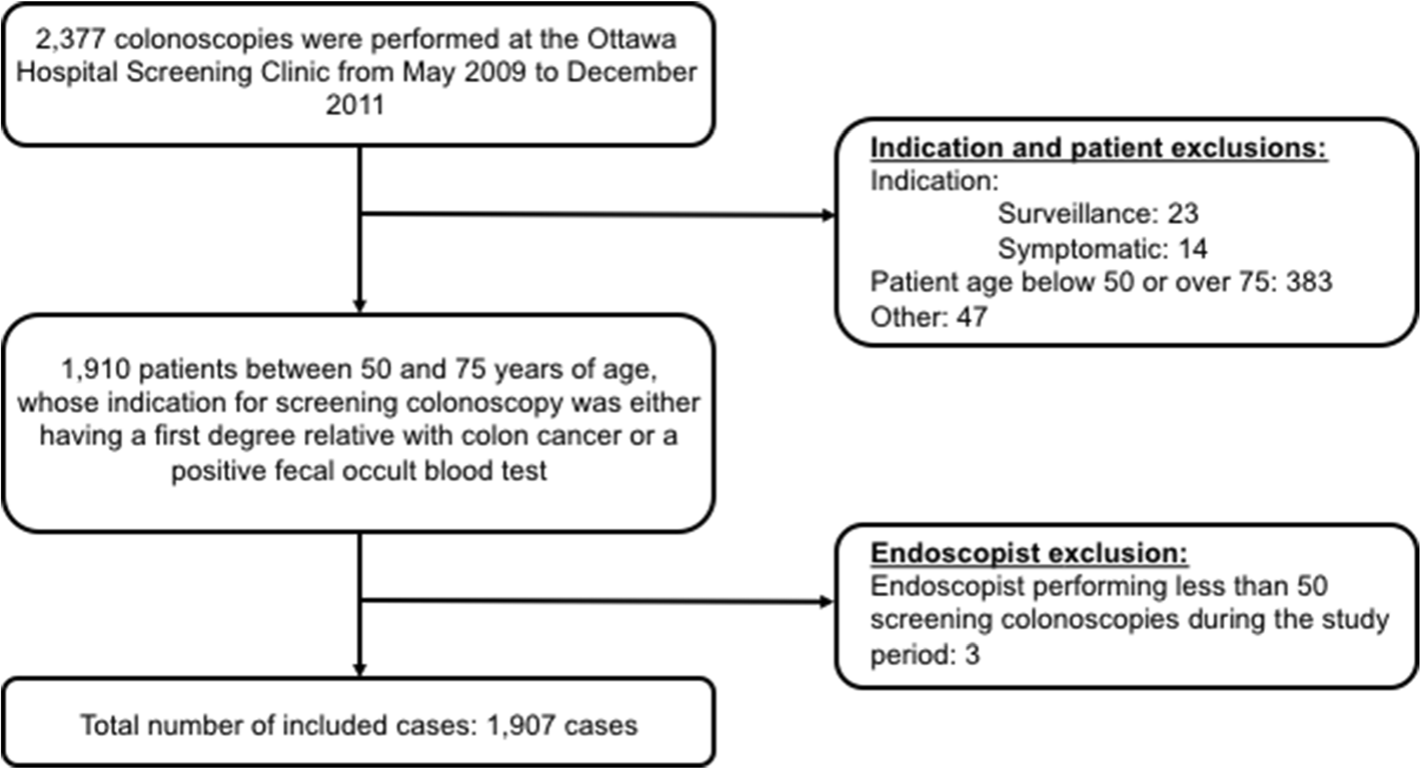
Data Inclusions Flowchart

A retrospective study was performed for all high definition white light colonoscopies between May 2009 and December 2011 at the Ottawa Hospital Colorectal Cancer Screening Clinic - a regional colon cancer screening center. Patients between 50 and 75 years of age, who were referred to the Colon Cancer Screening Clinic with a first degree relative with colon cancer or a positive fecal occult blood test (FOBT), were included. Colonoscopy Interim Reporting Tool (CIRT) forms were mandatorily filled out by the endoscopist of the screening clinic after each procedure. In these forms, procedure indication options included: symptoms, surveillance, positive FOBT, family history of colon cancer in a first-degree relative, and other. The indications of interest in this study included having a family history in a first-degree relative and a positive FOBT. Withdrawal time was not captured. Colonoscopies performed for other indications, including symptoms and colon cancer surveillance, were excluded. Cases where the endoscopist performed less than 50 screening colonoscopies during the study period were also excluded (Fig. 1).

Using the information from the CIRT data collection forms and patient chart review, a database was created containing patient (age and sex), endoscopist (specialty and number of years in practice), and procedure (indication for colonoscopy, bowel preparation, number of polyps found, and the location of each polyp in the colon) information. All bowel preparations involved 4 L of polyethylene glycol 3350 (Colyte). In the majority of cases, 2 L split prep dosing was used at the discretion of the endoscopist. Bowel preparation was defined as good when all residue was liquid and over 90% of the mucosal surface was visible, fair when there was some semisolid stool that could be suctioned or washed away and over 90% of the mucosa surface was visualized, and poor when semisolid stool could not be suctioned or washed away and less than 90% of the mucosal surface was visualized [15]. Cases involving good and fair bowel preparation were grouped together, as they have been shown to be associated with similar levels of adenoma detection [15].

Pathology reports were reviewed to determine histology of each colonic polyp removed. Consistent with previous studies [16], all polyps with adenoma or sessile serrated pathology were included as adenomas.

At our center, colonoscopy procedures are scheduled into 30-min time slots. To account for cases where 30 min may have been insufficient time to remove all of the polyps identified during the initial colonoscopy, we determined if an additional colonoscopy was performed within 12 months of the first (index) screening colonoscopy with the use of the Ottawa Hospital Colonoscopy Database. If additional colonoscopies were found, the number, location and histology of each polyp removed within one year of the index colonoscopy were collected and pooled for each patient. We also used this database to calculate each endoscopist’s colonoscopy case volume for the period of October 1st 2011 to March 31st 2012.

Each endoscopist was contacted directly in order to obtain information regarding their practicing specialty and their completion of training. This was then corroborated using the College of Physicians and Surgeons of Ontario’s public register.

The distribution of colonic polyps was assessed using the following definitions:

Distal colon = splenic flexure, descending colon, sigmoid, and rectum.

Proximal colon = cecum, ascending colon, hepatic flexure, and transverse colon.

ADR = proportion of colonoscopies where at least one adenoma is found based on histological analysis.

Distal ADR = proportion of colonoscopies where at least one adenoma is found in the distal colon.

Proximal ADR = proportion of colonoscopies where at least one adenoma is found in the proximal colon.

Adenoma to polyp detection rate quotient (ADPRQ) = proportion of removed polyps found to be adenomas on histological analysis.

### Statistical analyses

Categorical variables were reported as frequencies and proportions while continuous variables were presented as medians with interquartile range. Scatter plot and Spearman rank correlation were used to evaluate the association between ADR, distal ADR, and proximal ADR. Logistic regression models were developed to study univariate and multivariate associations between the independent variables and overall, distal, and proximal AD. Only those variables found to have a significant univariate association (*p*-value cut-off of < 0.05) with the dependent variables were included in the multivariate models. The data was analyzed using STATA statistical software version 13.1 (StataCorp, College Station, Texas, 2014).

## Results

A total of 1907 patients underwent screening colonoscopies and 11 endoscopists were included in the study (Table 1). The indication for the majority of patients was having a first degree relative with colon cancer (63%). The mean age was 60 years old and 42% of patients were male. In most cases (83%) the endoscopist was a gastroenterologist. Very few of the procedures involved poor bowel preparation (1%). The cecum was intubated in almost all of the cases (97%). More than one colonoscopy was performed within 12 months of the index colonoscopy in 70 patients (4%). Patient demographics and procedure details are presented in Table 1.

**Table 1:** Cohort demographics and procedure details for the entire cohort, *n* = 1907, and patients found to have at least one adenoma on colonoscopy

	Total Cohort (*N* = 1907)	Min. one adenoma (*N* = 467)	Min. one proximal adenoma (*N* = 248)	Min. one distal adenoma (*N* = 308)
Age, *Median (IQR) years*	60 (55–65)	61 (56–66)	62 (56–67)	61 (55–66)
Male Sex*, n(%)*	769 (41.7)	240 (51.4)	128 (51.6)	144 (52.0)
Indication, *n(%)*				
*Family history*	1198 (62.8)	263 (56.3)	148 (59.68)	138 (49.8)
*Positive FOBT*	709 (37.2)	204 (43.7)	100 (40.3)	139 (50.2)
Poor Bowel Preparation, *n(%)*	27 (1.4)	5 (1.1)	3 (1.2)	2 (0.7)
Colonoscopy Volume^a^, *Median (IQR)* *procedures*	150 (53–215)	166 (96–215)	174 (147–215)	157 (89–215)
Cecal intubation, *n(%)*	1857 (97.4)	559 (98.3)	244 (98.4)	273 (98.6)
Endoscopist Years of Practice, *Median (IQR)* *years*	26 (22–38)	23 (9–38)	21 (9–32)	24 (9–38)
Endoscopist Specialty, *n(%)*				
*Gastroenterology*	1580 (83.0)	356 (76.2)	212 (76.5)	181 (73.0)
*Surgery*	327 (17.2)	111 (23.8)	65 (23.5)	67 (27.0)

^a^Case volume over a 6 month period (October 1st 2011 to March 31st 2012)

Note: *IQR* interquartile range

### Adenoma detection

The overall ADR, distal ADR, and proximal ADRs were 25% (30% for males and 21% for females), 15, and 13%, respectively. The overall adenoma to polyp detection rate quotient (APDRQ) was 50%. Distal ADPRQ was 38% and proximal APDRQ was 63%. The median ADR, distal ADR, and proximal ADRs were 28% (interquartile range [IQR] 20–30%), 15% (IQR 11–20%), and 15% (IQR 10–19%), respectively.

### Proximal, distal, and overall inter-endoscopist ADR

**Fig. 2:**
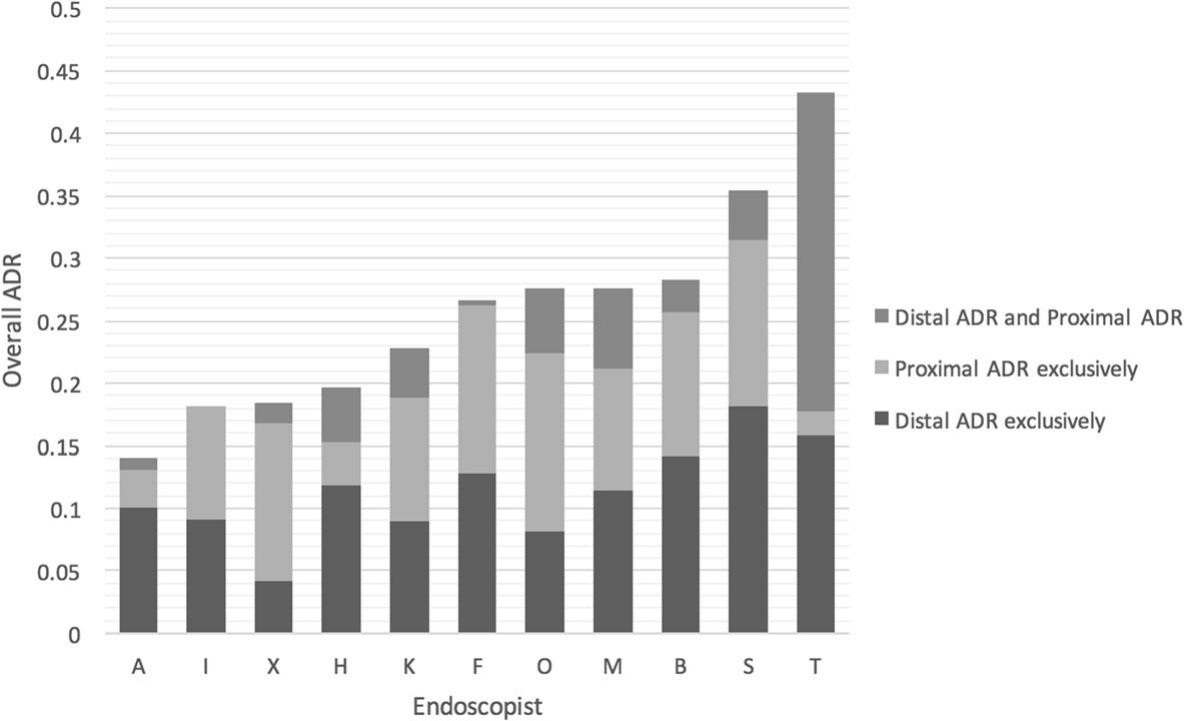
Adenoma Detection of Each Endoscopist. *Proximal ADR Exclusively = cases where ADR is solely made up of proximal ADR. Distal ADR Exclusively = cases where ADR is solely made up distal ADR

Figure 2 presents the overall ADR for each endoscopist as well as the proportion of their ADR that is attributed to adenoma detection in the proximal and distal colon. There was notable variability in both proximal and distal adenoma detection among endoscopists. Distal adenoma detection remained inconsistent even among higher performance endoscopists with overall ADRs greater than 25%.

### Correlation between endoscopist overall, distal, and proximal ADR

**Fig. 3:**
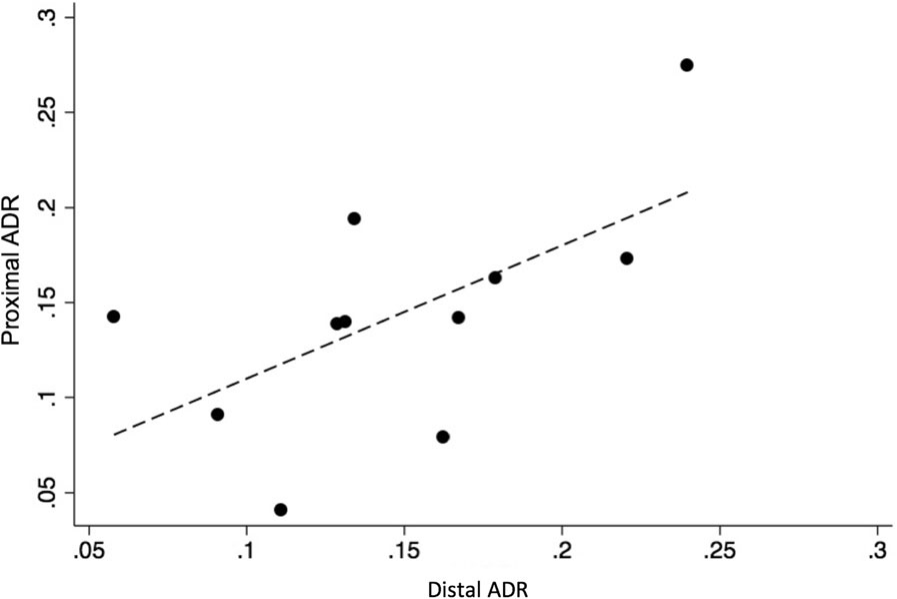
The association between proximal ADR and distal ADR

Intra-operator differences in distal vs proximal ADR were observed. Two of the endoscopists who had above the median proximal ADR, had below the median distal ADR, while the opposite was observed for two of the other higher performance endoscopists. The highest overall ADR (ADR range 29 to 45%) was found for the four endoscopists whose distal and proximal ADRs were above the median. The Spearman rank correlation (S) between distal and proximal ADR was 0.51 (*P* = 0.11), suggesting only a moderate positive association (Fig. 3). Overall ADR was found to be strongly associated with both distal (S = 0.78, *p* < 0.01) and proximal ADR (S = 0.83, *p* < 0.01).

### Factors associated with adenoma detection

Univariate logistic regression (Table 2) showed that overall adenoma detection was significantly associated with patient age (1.03 [95% CI: 1.01–1.05]), sex (1.68 [95% CI: 1.36–2.07]), endoscopist specialty (1.77 [95% CI: 1.37–2.29]) and years of practice (0.97 [95% CI: 0.97–0.98]). Distal and proximal adenoma detection were also significantly associated with these factors. Endoscopist colonoscopy volume was associated with overall and proximal, but not distal adenoma detection. Having a positive FOBT was associated with overall and distal, but not proximal adenoma detection (Table 2).

**Table 2:** Univariate Analysis for Predictors of Overall, Proximal, and Distal

	Overall AD			Proximal AD			Distal AD		
	OR	95% CI	*p*-value	OR	95% CI	*p*-value	OR	95% CI	*p*-value
Age:	1.03	1.01–1.05	< 0.01	1.04	1.02–1.07	< 0.01	1.02	1.00–1.04	0.01
(per year)									
Sex:	1.68	1.36–2.07	< 0.01	1.58	1.21–2.07	< 0.01	1.62	1.26–2.10	< 0.01
Male vs									
Female									
Indication:	1.44	1.16–1.78	< 0.01	1.16	0.89–1.53	0.27	1.89	1.45–2.42	< 0.01
Family history vs									
Positive FOBT									
Bowel preparation:	0.67	0.26–1.85	0.47	0.83	0.25–2.80	0.77	0.47	0.11–1.98	0.30
Poor vs									
Good or fair									
Colonoscopy volume	1.00	1.00–1.00	< 0.01	1.00	1.00–1.01	< 0.01	1.00	1.00–1.00	0.15
(per procedure)									
Intubation:	1.72	0.80–3.70	0.16	1.74	0.62–4.88	0.29	1.98	0.48–2.19	0.19
Cecal vs									
Incomplete									
Years of practice	0.97	0.97–0.98	< 0.01	0.96	0.95–0.97	< 0.01	0.98	0.71–5.55	< 0.01
(per year)									
Endoscopist Specialty:	1.77	1.37–2.29	< 0.01	2.00	1.46–2.72	< 0.01	1.60	1.18 - 2.18	< 0.01
Gastroenterology vs									
Surgery									

Note: *AD* Adenoma detection

In multivariate analyses, age, sex, and endoscopist years of practice remained significantly associated with overall, distal, and proximal adenoma detection. While increased age and male sex were associated with higher adenoma detection, greater endoscopist years of practice was related to lower adenoma detection. The association between adenoma detection and endoscopist specialty was no longer significant (Table 3).

**Table 3:** Multivariate Analysis for Predictors of Overall, Proximal and Distal

	Overall AD		
	OR	95% CI	*p*-value
Age (per year)	1.03	1.02–1.05	< 0.01
Sex Male vs Female	1.59	1.29–1.95	< 0.01
Indication Family history vs Positive FOBT	1.31	1.06–1.63	0.01
Colonoscopy volume (per procedure)	1.00	1.00–1.00	< 0.01
Years of practice (per year)	0.98	0.97–0.99	< 0.01
Endoscopist Specialty Gastroenterology vs Surgery	1.26	0.90–1.76	0.18
	Proximal AD		
	OR	95% CI	*p*-value
Age (per year)	1.05	1.03–1.07	< 0.01
Sex Male vs Female	1.65	1.25–2.17	< 0.01
Colonoscopy volume (per procedure)	1.01	1.00–1.01	< 0.01
Years of practice (per year)	0.96	0.95–0.98	< 0.01
Endoscopist Specialty Gastroenterology vs Surgery	1.13	0.73–1.74	0.58
	Distal AD		
	OR	95% CI	p-value
Age (per year)	1.02	1.00–1.04	0.03
Sex Male vs Female	1.52	1.17–1.97	< 0.01
Indication Family history vs Positive FOBT	1.68	1.30–2.18	< 0.01
Years of practice (per year)	0.99	0.97–1.00	0.035
Endoscopist Specialty Gastroenterology vs Surgery	1.15	0.77–1.74	0.49

Note: *AD* Adenoma detection

## Discussion

In this study, we demonstrate a notable variability in endoscopist distal and proximal adenoma detection. Our findings suggest that distal and proximal ADR, used in conjunction with overall ADR, can improve the assessment of colonoscopy quality.

The observed variability in adenoma detection rates between the distal and proximal colon may be attributable to both physiological differences between colon segments and the varibility in operator skill sets. For instance, there are reported differences in segment-specific adenoma occurrence rate [13, 17]. Boroff and colleagues found that while only 30% of distal polyps are diagnosed as adenomas on histology, adenomas correspond to 76% of polyps in the proximal colon [13]. Consistent with these findings, we observed markedly lower distal ADPRQ, as compared to proximal APDRQ (38 and 63%, respectively).

Adenoma detection is also dependent on skill, training and experience, as demonstrated by significant variability in ADR between endoscopists [18]. Interestingly, adenoma miss rates between endoscopists have been previously found to differ for the distal and proximal colon, suggesting that different skills may be required for these segments [12]. In accordance with this observation, our study demonstrates that proficiency in distal adenoma detection does not directly correlate with proximal adenoma detection for the same endoscopist. This observation was independent of endoscopist overall performance (as indicated by overall ADR). Section-specific operator skills necessary for adenoma detection may relate to variances in polyp morphology [19], frequency [10], and the extent of blind spots in certain parts of the distal and proximal colon [20].

Some specific operator skill measures found to be associated with overall ADR include endoscopist accreditation and colonoscopy volume [21], as well as years of experience [22]. Here we confirm that overall ADR is correlated with all of these operator characteristics. However, while proximal adenoma detection was associated with endoscopist procedure volume, that was not the case for distal ADR. This indicates that the increase in ADR among higher volume endoscopists may be related to their ability to detect adenomas in the proximal colon. Endoscopists with a higher degree of skill and experience are more likely to complete the colonoscopy to the cecum and are better attuned to detecting sessile serrated adenomas [23]. In the setting of limited endoscopy time, if more time is attributed to identifying and removing polyps the proximal colon, the withdrawal time for the distal colon may be compromised, resulting in reduced distal adenoma detection [24].

Adenoma detection also showed a weak inverse association with endoscopy years of practice. This is consistent with previous research demonstrating that optimal ADR occurs with a lower number of years of experience [15], suggesting that training plays a more important role in the quality of adenoma detection than years of experience [25].

Patient-related overall ADR determinants are known to include patient age and gender [26]. Our findings confirm these observations and demonstrate similar associations for both distal and proximal ADR.

Certain study limitations should be noted. First, the study was carried out in a single institution setting. Future investigations should determine if our observations are applicable elsewhere. On the other hand, our observed variability in proximal and distal adenoma detection is consistent with what has been previously observed [14]. Second, we were unable to examine certain procedural factors that have been previously associated with ADR such as withdrawal time [27] and time of day [28, 29]. The influence of colonoscopy queue position during the day, insertion time, and withdrawal time on proximal and distal adenoma detection warrant further study. Finally, we did not differentiate between sessile and non-sessile adenomas. Sessile polyps have been shown to be more difficult to detect and may represent higher risk lesions [30, 31]. However, our approach is consistent with the existing endoscopy quality literature, which includes sessile serrated polyps as adenomas [15]. Finally, the overall ADR for this study was 25% which, although within the acceptable quality standards for ADR recommended by the American Society for Gastrointestinal Endocopy and the American College of Gastroenterology [1, 5], are lower than ADRs presented elsewhere. We believe that the two main factors contributed our observed lower ADR: the high number of years in practice and low median annual volume of the practicing endoscopists. Both of these factors were associated with lower ADR in this study. This may limit the generalizability of our results and future research should determine whether our findings are reproducible in other centres.

For future investigations, we also recommend the analysis of advanced technologies such as EndoCuff, EndoRings, G-EYE™, FUSE, and cap-assisted colonoscopy. These techniques have been related to ADR rates of up 69%. It has been proposed that using these devices to flatten folds and improving endoscope stability can lead to narrowing the adenoma detection differences between the distal and proximal colon [32], however these technologies may be more applicable to augmenting ADR is specific areas of the colon. It has been previously demonstrated that feedback regarding overall ADR results in improvement in proximal but not distal adenoma detection [33]. An additional question of interest would be whether colon segment-specific performance feedback to colonoscopists can produce a more even distribution of improvement.

## Conclusions

We found notable variability in endoscopists’ distal and proximal adenoma detection. While distal ADR and proximal ADR are both associated with increased overall ADR, endoscopist distal ADR was only moderately correlated with their proximal ADR. Information regarding both distal ADR and proximal ADR may complement currently used quality indicators to provide endoscopists with informative feedback regarding their adenoma detection proficiency.

## Abbreviations

ADR: Adenoma Detection Rate
APDRQ: Polyp detection rate quotient
CIRT: Colonoscopy Interim Reporting Tool
CRC: Colorectal Cancer
FOBT: Fecal occult blood test
S: Spearman rank correlation
